# Comparative biological activity of abamectin formulations on root-knot nematodes (*Meloidogyne* spp.) infecting cucumber plants: in vivo and in vitro

**DOI:** 10.1038/s41598-023-39324-x

**Published:** 2023-07-31

**Authors:** Magdy A. Massoud, Abdel Fattah S. A. Saad, Mohamed S. Khalil, Mosher Zakaria, Shady Selim

**Affiliations:** 1 Plant Protection Department, Faculty of Agriculture (Saba-Basha), Alexandria University, Alexandria, 21531 Egypt; 2 Fungicides, Bactericides and Nematicides Department, Central Agricultural Pesticides Laboratory, Agricultural Research Center, El-Sabheya, Alexandria, Egypt; 3 Faculty of Desert and Environmental Agriculture, Department of Pesticide Chemistry and Technology, Matrouh University, Matrouh, Egypt

**Keywords:** Plant sciences, Zoology

## Abstract

The root-knot nematodes (*Meloidogyne* spp.) are considered one of the most destructive diseases in the world. In Egypt, farmers primarily rely on chemical nematicides, which have become costly to control. Currently, abamectin is a bio-based pesticide used as an alternative tool against *Meloidogyne* spp. on cucumber plants (*Cucumis sativus* L.). During the current research, four tested abamectin formulations were DIVA (1.8% EW), RIOMECTIN (5% ME), AGRIMEC GOLD (8.4% SC) and ZORO (3.6% EC) compared with two reference nematicides namely, CROP NEMA (5% CS) and TERVIGO (2% SC). The main results showed that, in vitro study elucidated that the most effective formulations of abamectin as a larvicidal were EW with LC_50_ value of 21.66 µg ml^−1^. However, in the egg hatching test, the formulations of abamectin SC (2%) and EW were the most effective in reducing egg hatching, with LC_50_ values of 12.83 and 13.57 µg ml^−1^. The calculated relative potency values showed diversity depending on the two referenced nematicides. On the other hand, in vivo study, the results indicated that, all tested formulations of abamectin recorded general mean reductions in root galls (23.05–75.23%), egg masses (14.46–65.63%). Moreover, the total population density declined by 39.24–87.08%. Furthermore, the influence of abamectin formulations, in the presence of root-knot nematodes, on the growth of cucumber plants parameters, such as root dry weight, root length, root radius, root surface area, shoot dry weight and shoot height, as well as the content of macro-elements (N, P and K) exhibited varying levels of response.

## Introduction

Root-knot nematodes RKNs (*Meloidogyne* spp.) are one of the most destructive pests globally and cause great economic losses in agricultural crops due to their wide host range and variety of suitable climates^1^. Otherwise, plant parasitic nematode (PPN) amplifies the sensitivity and susceptibility of major host plants to be attacked by biotic stresses such as fungi and bacteria, leading to economical yield loss of 5–12% per year. Many crops may become more sensitive to PPN once it’s emerges in the agroecosystem^2^. It is expected to cause an annual global agricultural loss of $78 billion^3^.

Cucumber plants (*Cucumis sativus* L.) belong to the Cucurbitaceae family, and they can be grown in home gardens, open fields or in greenhouses in cool climates. Cucumber fruit contains silicon, potassium, sulphur, sodium, and acid, creating materials that are helpful to maintain the human blood’s alkalinity. Also it contains fiber, manganese, magnesium, and vitamins K, C and A. also have antimicrobial and anticancer properties, as well as detoxify the body and prevent some bone diseases^4^. Moreover, cucumber is one of the economically important vegetables cultivated in greenhouses in Egypt. However, in 2019 Egypt produced 364,571 tons with the harvested area estimated at about 16,104 ha^5,6^. On the other hand cucumber is one of the famous and favorite hosts to the RKNs which reduces the content of chlorophyll, amino acids and organic acids in the plant, causing a 25% yield loss annually in open fields^7^.

Nematicides are chemicals employed in various agricultural practices to control plant parasitic nematodes. Farmers are preferred to use non-fumigant nematicides, especially traditional nematicides^8^. The available choices to manage plant parasitic nematodes are limited. Nowadays, abamectin is one of these available choices^9^.

Abamectin is a 16-membered macrocyclic lactone family of avermectins that includes doramectin, ivermectin and selamectin which are produced by the fermentation process of a gram-positive bacterium, *Streptomyces avermitilis* and have acaricidal, insecticidal, and nematicidal effects^10–13^. It was introduced in 1985 as an acaricide, insecticide and nematicide with contact and stomach action by Syngenta^14^. Also, it’s a blend of avermectin ≥ 80% B1_a_ and ≤ 20% B1_b_, these two components have similar the toxicological and biological effects^15^.

Moreover, abamectin’s mode of action works on electrical conductivity of the neuronal cells by blocking the transmission of electric pulses by binding of gamma-amino butyric acid (GABA) at the nerve terminals^16,17^. As a result, a glutamate-gated chloride channels are activated and opened to allow the influx of chloride ions into the cell, causing hyperpolarization consequently, paralyzing the neuromuscular system and death^18,19^.

In addition, abamectin has no carcinogenic, teratogenic, or mutagenic effects on mice or other mammals, and only very high concentrations produce semi-lethal toxicity^20^. However, the semi-lethal concentrations are rarely occur in the environment, while accumulation at low rates over a long period of time could be highly toxic to fish and consequently could enter the human body as a part of the biological food chain^21^. Otherwise, abamectin could be highly toxic if inhaled or swallowed, and in addition, patients could recover with treatment, although, sub-chronic and chronic toxic effects are still unclear for low-dose and long-term exposure^20^.

Many studies indicated that abamectin has been extensively reported and registered to control the root-knot nematodes (RKN)^1,22,23^. However, abamectin have 110 registered solo products in Egypt, divided into: 85 emulsifiable concentrates (EC), 17 suspension concentrates (SC), four micro emulsions (ME), two emulsion-in-water (EW), and two capsule suspensions (CS) formulations. However, only two products with different formulations registered as nematicides TERVIGO (2% SC) and CROP NEMA (5% CS)^24^. Due to the serious lack of nematicides in Egypt and the favorable of abamectin as a bio-based nematicide it raised a question that does the other formulation could be used as a nematicides beside the recommended products?, therefore, the current study aimed to select one or more of different local registered abamectin formulations in the Egyptian markets and investigate their—nematicidal performance and potency against root-knot nematodes (*Meloidogyne* spp.) and plant growth parameters to determine which formulation could be recommended for future prospects in in vitro and in vivo experiments.

## Materials and methods

### Root-knot nematode inocula

The source of the root-knot nematodes (*Meloidogyne* spp.) culture was originally isolated from the root of cucumber (*Cucumis sativus* cv. Dahab) transplanted under plastic house conditions at the Agricultural Research Center (ARC), El-Sabheya, Alexandria, Egypt. The collected plant samples were taken from the plastic house designated for scientific research. The eggs of the root-knot nematode were extracted from roots by sodium hypochlorite (NaOCl) according to^25^, while the second stage juveniles (*J*_*2*_) were obtained from the hatched eggs by Baermann plate technique^26^.

### The tested abamectin formulations and their rates

Six different commercial formulations of abamectin products were selected from the Egyptian markets to be investigated under in vitro and in vivo conditions. The abamectin formulations were DIVA (1.8% EW), RIOMECTIN (5% ME), AGRIMEC GOLD (8.4% SC), ZORO (3.6% EC), CROP NEMA (5% CS) and TERVIGO (2% SC). Noteworthy, there were two SC formulations; the 1st was AGRIMEC GOLD (8.4% SC) and the 2nd was the referenced nematicide TERVIGO (2% SC). Each product was evaluated in two rates: 50 and 100 g a.i./feddan; these rates were established according to the standards registered in Egypt.

### In vitro assays

The impact of different abamectin formulations on egg hatching and larval mortality of the root-knot nematodes (*Meloidogyne* spp.) was assessed under laboratory conditions (29 ± 2 °C). Moreover, several experiments were conducted to establish the effective concentration ranges of abamectin products.

#### Hatching assays

The tested concentrations of abamectin products were ranged from 2.5 up to 800 µg/ml. The vials (each one ca. 15 ml) containing distilled water served as untreated checks. Each concentration was replicated four times and each replicate included approximately 1000 eggs. The numbers of hatched eggs were recorded and their EC_50_ were calculated after 7 days of application.

#### Mortality assays

The tested concentrations of each abamectin product during this study ranged from 2.5 up to 800 µg/ml. Each concentration was replicated four times, and each replicate included approximately 200 *J*_*2*_*s*. Vials included distilled water served as controls. The numbers of both dead and alive J_2_ were recorded after 48 h of exposure, and the mortality percentages were calculated.

### In vivo assays (experimental design of microplots)

The performance of abamectin products was investigated on cucumber plants infested with root-knot nematodes. The pots were filled with 2 kg of autoclaved sandy soil (pH 8.3, O.M. 0.18%). All abamectin formulations were applied as soil drench at two rates of 50 and 100 g a.i./feddan in comparison to two standard products, namely, CROP NEMA (5% CS) and TERVIGO (2% SC). A cucumber plantlet (cv. Dahab) was transplanted into each pot, and 3 days later the inoculation process with 7000 eggs/pot was executed. Two types of untreated controls were used: inoculated (Control +) and uninoculated (Control −).

All treatments were replicated five times, and pots were in outdoor conditions (28 ± 2 °C, 70 ± 2 RH and 14: 10 lights: dark period). During the experiment, irrigation and fertilization were applied when appropriate. Sixty-two days later, after the inoculation, the plants were removed and washed to be free of soil. Shoot height, shoot dry weight and root dry weight, in addition to the root length, root radius and root surface area^27^ were estimated. The second stage of juveniles (*J*_*2*_*s*) were extracted, and the roots were stained with Phloxine B to facilitate egg mass counting. The number of galls/2 g roots and egg-masses/2 g roots were counted. The total population density was estimated by quantifying and summing individuals of eggs/root system together with the second stage of juveniles/2 kg soil^28^. The macro-elements such as nitrogen (N), phosphorus (P), and potassium (K) were determined in the cucumber tissues at the termination of the experiment in the lab at the department of reclamation and cultivation of desert lands, faculty of Agriculture (Saba-Basha), Alexandria University. However, the cucumber used in this study (cv. Dahab) is formally registered in the Egyptian Ministry of Agriculture.

### Statistical analysis and experimental design

The hatching and *J*_*2*_*s* mortality percentages were estimated using the Abbott formula^29^, and Probit analysis was used to calculate the LC_50_ for larvae and EC_50_ for eggs each compound according to^30^. The relative potency of tested products was calculated according to^31^ using the Polo plus program^32^ for two references (SC and CS formulations). The statistical analysis of data was carried out using the computer program^33^. The microplots experiment was arranged in a complete randomized (CRD) design with five replications for each treatment, each replicate consisted of one plant. Statistically, the significant differences between the means were compared using analysis of variance (ANOVA) with the least significant differences (LSD) and *P* values = 0.05 probability.

### Ethics approval and consent to participate

This article does not contain any studies with human or animal subjects. The current experimental research including the collection of plant material, is complying with relevant institutional, national, and international guidelines and legislation and used for research and development.

### Permission statement

To collect the plant material for this study a permission was obtained from Prof. El-sayed H. Eshra, the head of plant protection research station, Agricultural Research Center, Alexandria, Egypt. Also, The Agricultural Research Center who’s the responsibility for formal identification of the plant material used in our study.

## Results

### In vitro study of larval and egg hatching tests

**Figure 1:**
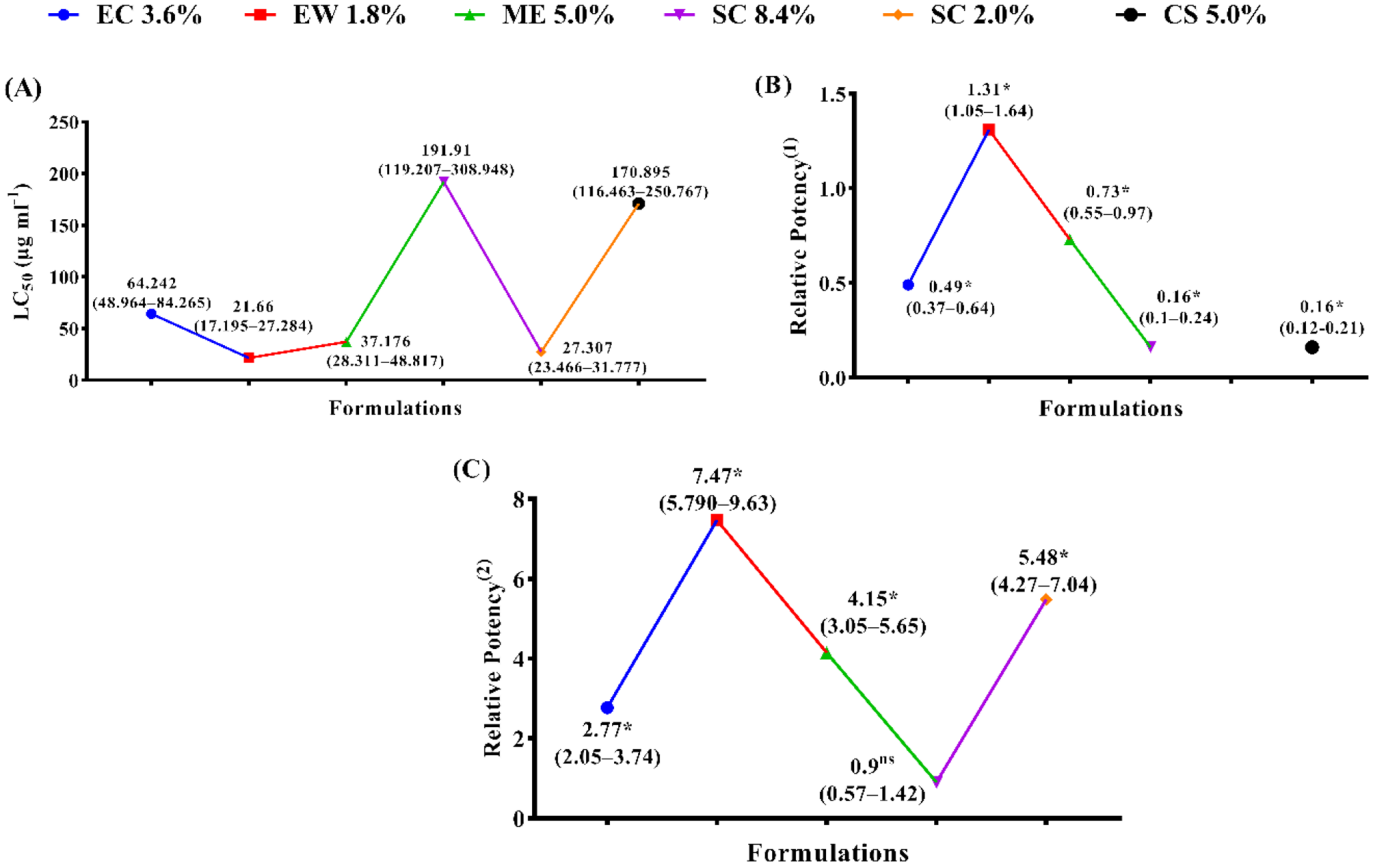
The larvicidal activity of various abamectin formulations, in vitro study, against the J_2_ of the root-knot nematodes (*Meloidogyne* spp.). (**A**) LC_50_ (µg ml^−1^) values (Fiducial Limits), (**B**) Relative potency^(1)^ values (Fiducial Limits), and (**C**) Relative potency^(2)^ values (Fiducial Limits). * Asterisks (*) means significant differences while (^ns^) means not significant. * (^1^) and (^2^) are referenced nematicides (TERVIGO and CROP NEMA, respectively).

The data in (Fig. 1A–C) presented the larvicidal activity of abamectin at various formulations against the second-stage juveniles (*J*_*2*_) of the root-knot nematodes after 48 h of exposure. The investigated formulations of abamectin were EC (3.6%), EW (1.8%), ME (5%) and SC (8.4%), in compared with two referenced nematicides formulated as SC (2%) and CS (5%). Results showed that the most effective formulations of abamectin were EW (1.8%), SC (2%), ME (5%), EC (3.6), CS (5%) and SC (8.4%) based on the calculated LC_50_ values of 21.66, 27.31, 37.18, 64.24, 170.90 and 191.91 µg/ml, respectively. The calculated values of relative potency were 1.31, 0.73, 0.49, 0.16 and 0.16 folds with EW, ME, EC, SC (8.4%) and CS formulations as compared with the referenced nematicide as SC (2%), respectively. Otherwise, the values of relative potency were 7.47, 5.48, 4.15, 2.77 and 0.90 folds with EW, SC (2%), ME, EC, and SC (8.4%) formulations when compared with the referenced nematicide as CS (5%), respectively.

**Figure 2:**
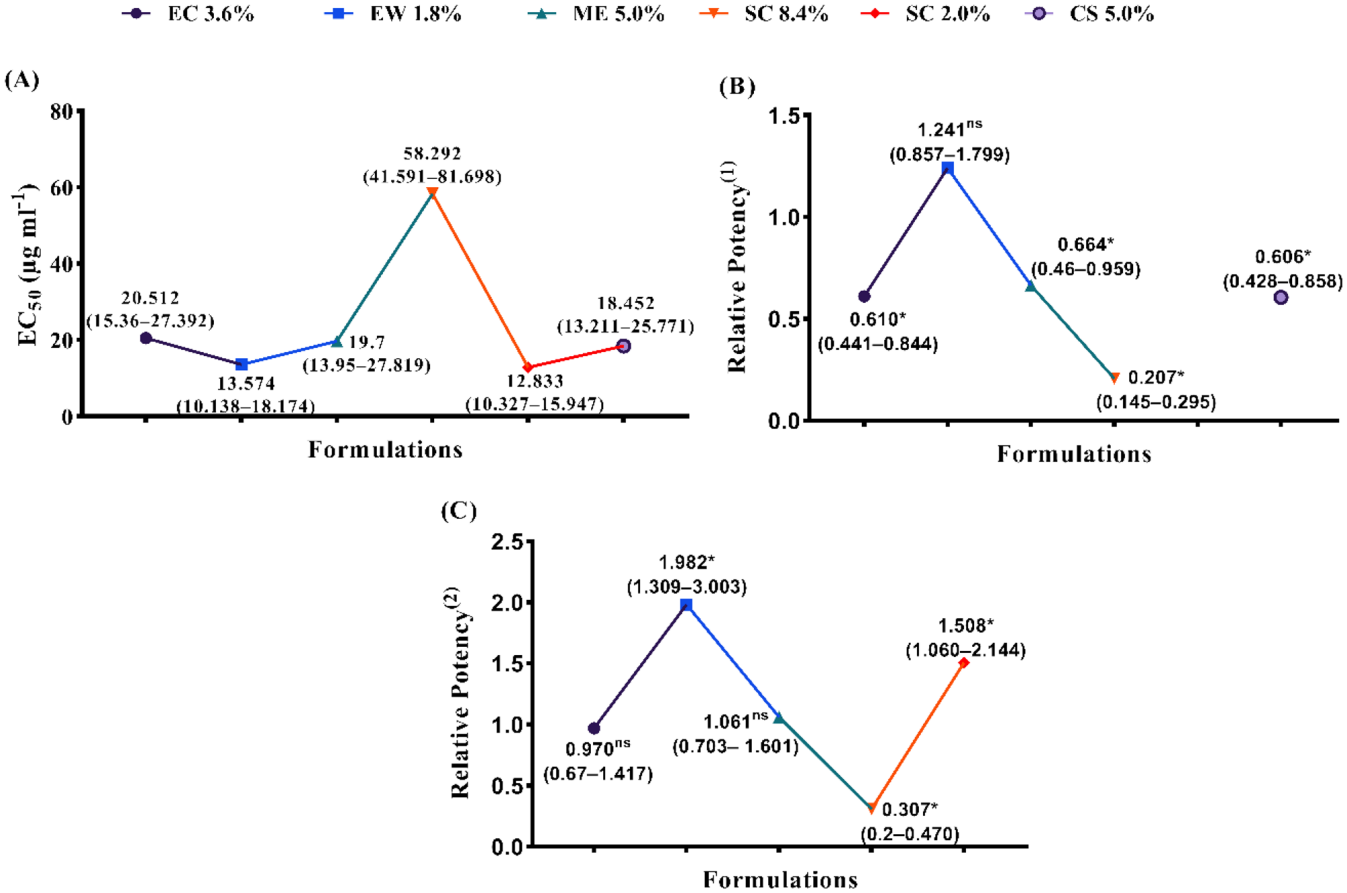
The egg hatching rate of various abamectin formulations, in vitro study against the J_2_ of the root-knot nematodes (*Meloidogyne* spp.). (**A**) EC_50_ (µg ml^−1^) values (Fiducial Limits), (**B**) Relative Potency^(1)^ values (Fiducial Limits) and (**C**) Relative Potency^(2)^ values (Fiducial Limits). * Asterisks (^*^) means significant differences while (^ns^) means not significant. * (^1^) and (^2^) are referenced nematicides (TERVIGO and CROP NEMA, respectively).

Regarding to effect on eggs hatching rate, the influence of different abamectin formulations namely, EC (3.6%), EW (1.8%), ME (5%) and SC (8.4%), was assessed against the eggs of the root-knot nematodes (*Meloidogyne* spp.) after 7 days of exposure under laboratory conditions (29 ± 2 °C), and compared to the two referenced nematicides at SC (2%) and CS (5%) (see Fig. 2A–C). The obtained data exhibited that the most effective formulations of abamectin to decrease the hatching rate of eggs were SC (2%), EW, CS, ME, EC, and SC (8.4%) with values of EC_50_ estimated by 12.83, 13.57, 18.45, 19.70, 20.51 and 58.29 µg/ml, respectively. The values of relative potency were 1.24, 0.66, 0.61, 0.61 and 0.21 folds with EW, ME, EC, CS, SC formulations when compared with the referenced nematicide at SC (2%), respectively. Also, the relative potency of EW, SC (2%), ME, EC, SC (8.4%) formulations in compared with the referenced nematicide at CS (5%) recorded values estimated by 1.98, 1.51, 1.06, 0.97 and 0.31 folds, consecutively. In general, these formulations could be arranged in a descending order according to their effectiveness on *J*_*2*_ mortality as follows: EW (1.8%) > SC (2%) > ME (5%) > EC (3.6%) > CS (2%) > SC (8.4%). In addition, these formulations could be arranged in descending order according to their effectiveness on egg hatching as follows: SC (2%) > EW (1.8%) > CS (5%) > ME (5%) > EC (3.6%) > SC (8.4%).

### Microplot experiment (in vivo study)

**Figure 3:**
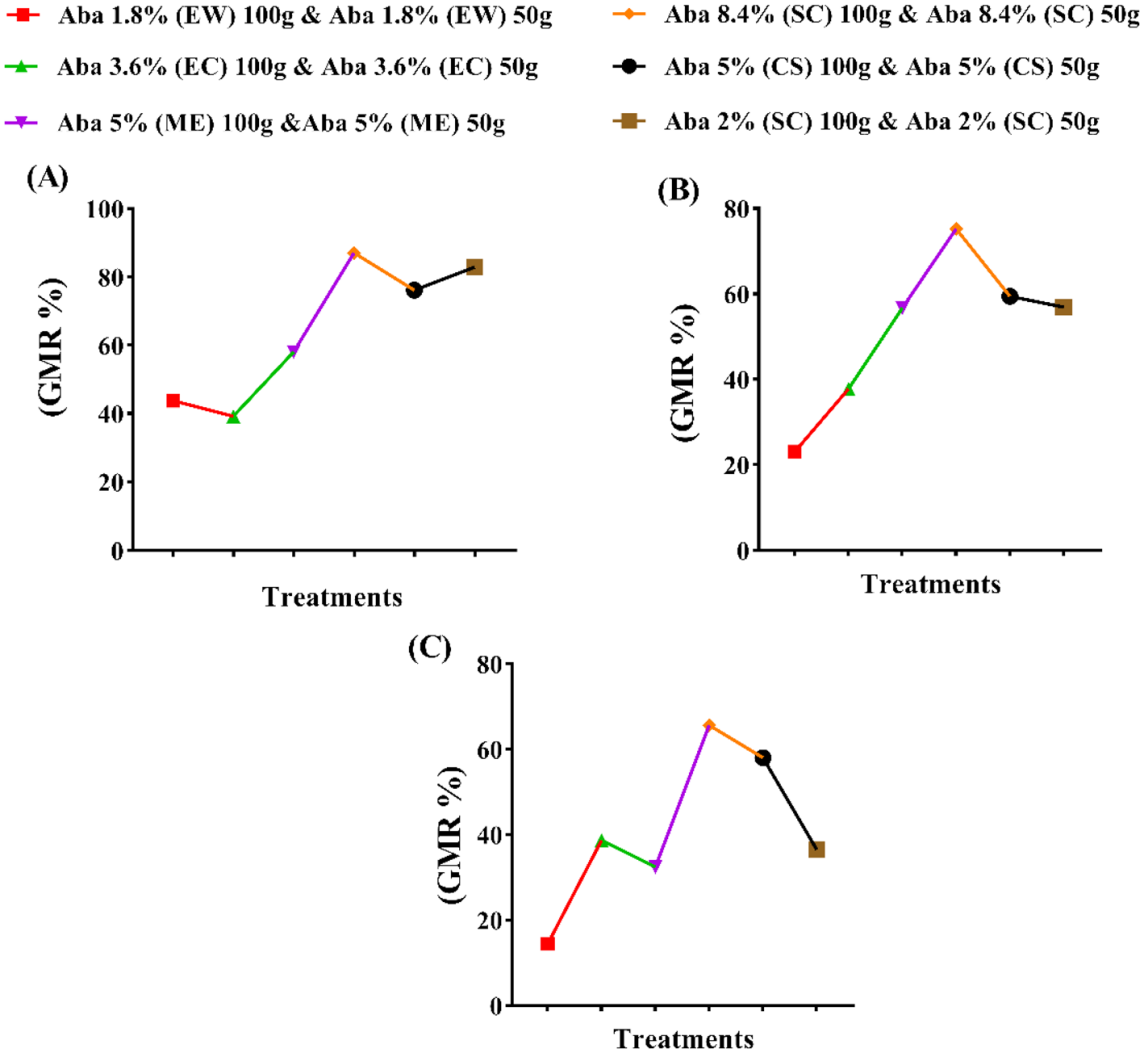
The efficacy of certain formulations of the abamectin at two rates as soil treatments against galls no., egg masses no. and the total population density of the root-knot nematode (*Meloidogyne* spp.) on cucumber plants. (**A**) General mean reductions (%) of total population density, (**B**) General mean reductions (%) of galls no./2g roots and (**D**) General mean reductions (%) of egg masses no. /2g roots. The general mean reduction percentage (GMR %) was calculated as (N/2), whereas N = Sum of reduction (Red. %) values of the two active dose rates (50g and 100g) of abamectin. While reduction (Red. %) was calculated as {(C^+^- T/C^+^) *100}, Whereas C+ = (Control +) value, T = (Treatment) value and in final, the total population density = (Total J_2_ No. per pot + total eggs number per pot)/1000.

**Figure 4:**
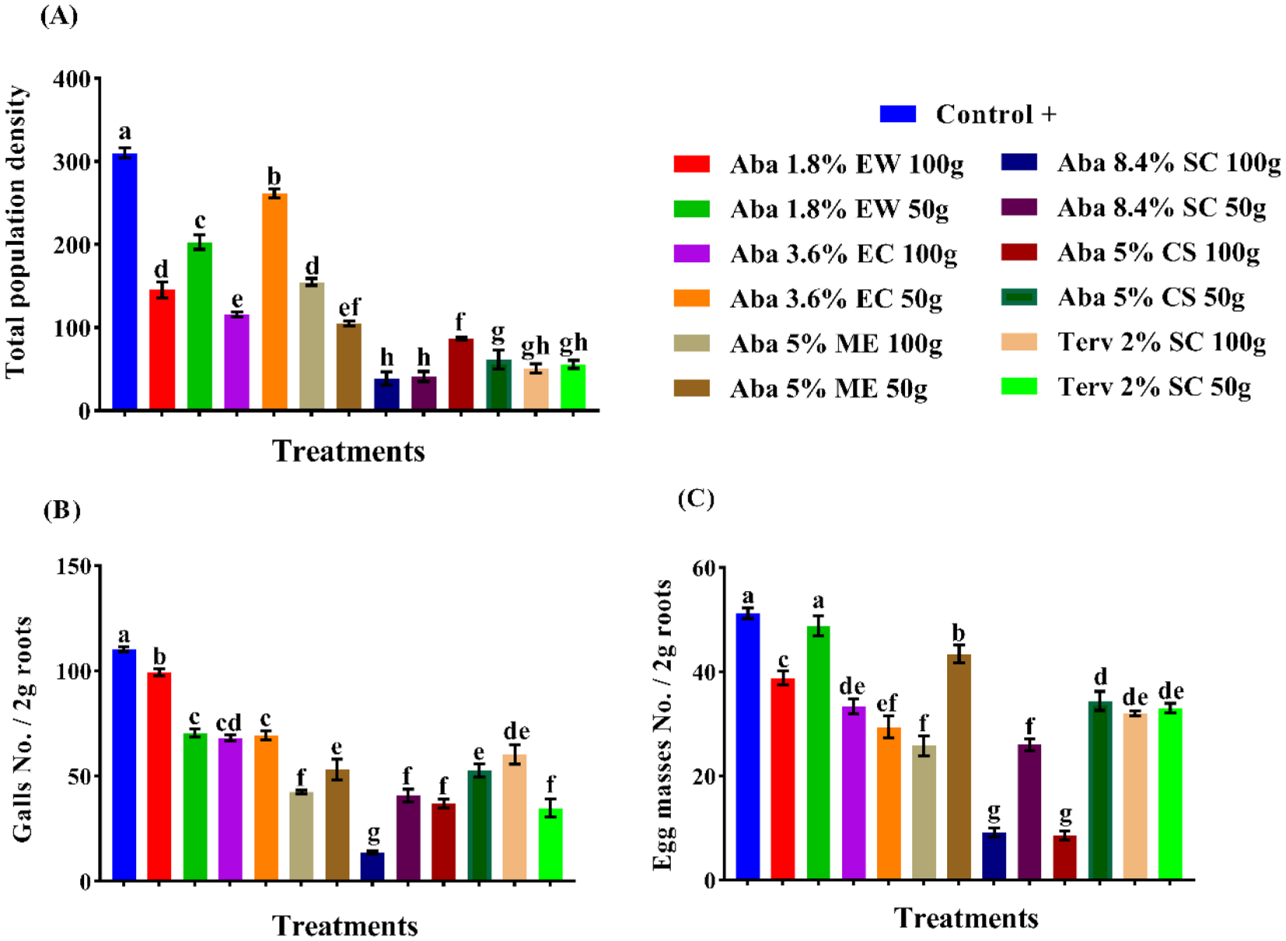
The efficacy of abamectin in various formulations and rates against *Meloidogyne* spp. on cucumber plants. (Means in each column followed by the same letter(s) are not significantly different according to LSD (*p* = *0.05*), values are means ± SEM).

The tested products of abamectin were evaluated at two rates 50 and 100g a.i./feddan. Also, both referenced nematicides; CROP NEMA (5% CS) and TERVIGO (2% SC) were used in comparison at both rates (Figs. 3A–C, 4A–C). The application of abamectin as SC (8.4%) recorded the highest general mean reduction (GMR%) in the total population density (J_2_ + eggs) of *Meloidogyne* spp. followed by SC (2%), CS, ME, EW, and EC with 87.08, 82.87, 76.11, 58.05, 43.82 and 39.24%, successively. The high rate of the formulations (100 g a.i./feddan) recorded the highest reduction percentages compared to the lowest rate (50 g a.i./feddan), except with ME and CS formulations. There are significant differences between the two tested rates except for SC (8.4%) and SC (2%) (Figs. 3A–C, 4A–C).

The results for gall formation were significantly suppressed with SC (8.4%), CS, SC (2%), ME, EC, and EW formulations by 75.23, 59.44, 56.90, 56.72, 37.75 and 23.05%, respectively. However, no significant difference was noticed between the high and low rates of EC formulation, while the remaining formulations showed significant differences (Figs. 3A–C, 4A–C).

The egg masses were decreased with all applied treatments (Figs. 3A–C, 4A–C). The application of abamectin as SC (8.4%), CS, EC, SC (2%), ME and EW formulations recorded GMR of 65.63, 58.01, 38.68, 36.53, 32.42 and 14.46%, successively. The significance analysis exhibited that no significant differences were observed between the high and low rates of EC, and SC (2%) formulations.

### The effect of abamectin formulations on cucumber growth

**Figure 5:**
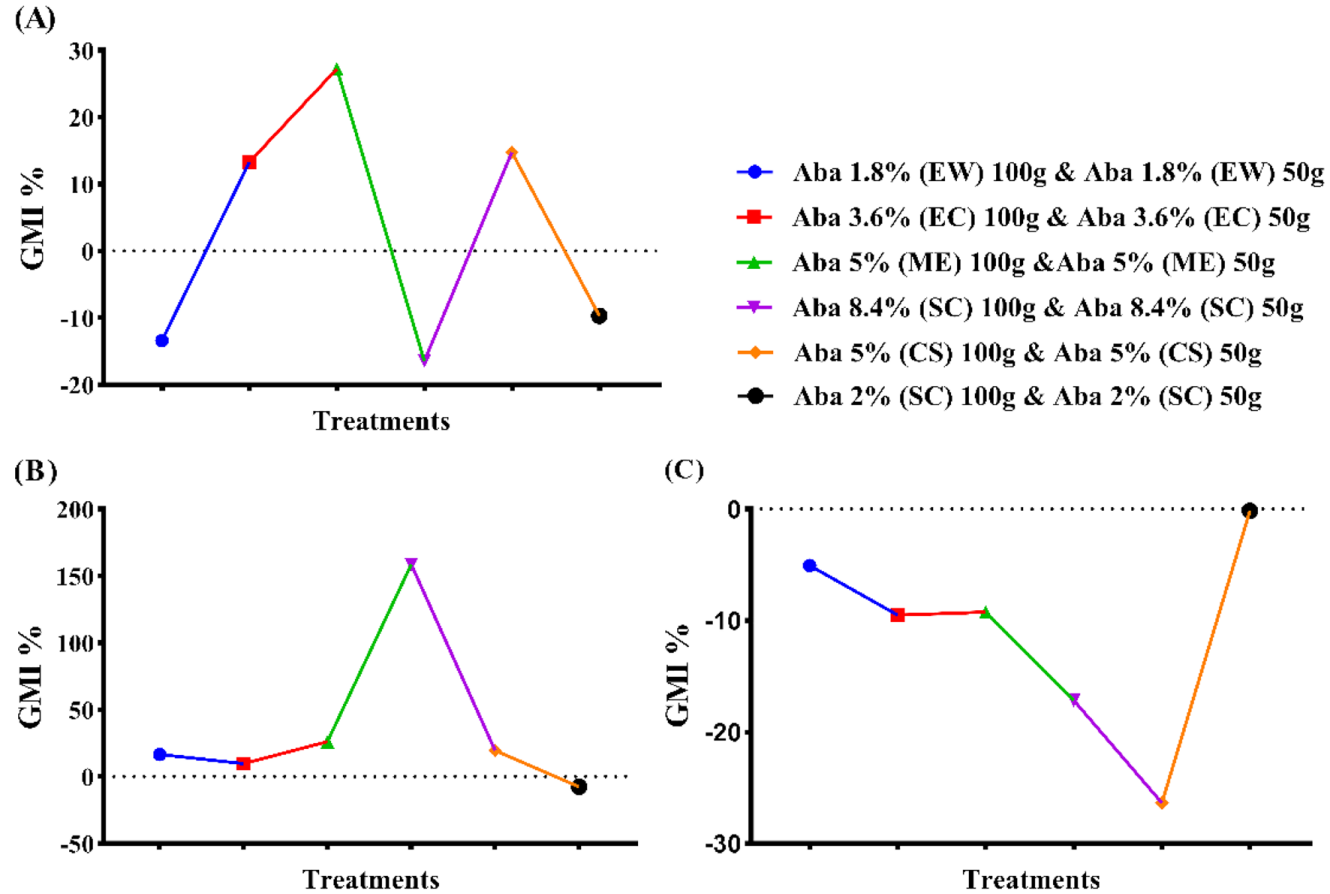
The influence of certain formulations of abamectin at two rates as soil treatments on the growth of cucumber plant infected by (*Meloidogyne* spp.). (**A**) General mean increases (%) of the root sys. dry weight, (**B**) General mean increases (%) of the shoot sys. dry weight and (**C**) General mean increases (%) of the shoot sys. height. The general mean increase percentage (GMI %) was calculated as N/2, whereas N = Sum of the increase percentage (I %) values of the two active dose rates (50 g and 100 g) of abamectin. Whereas I (%) = {(T − C^+^/C^+^) * 100} while, T: (Treatment) value and C^+^: (Control +) value.

**Figure 6:**
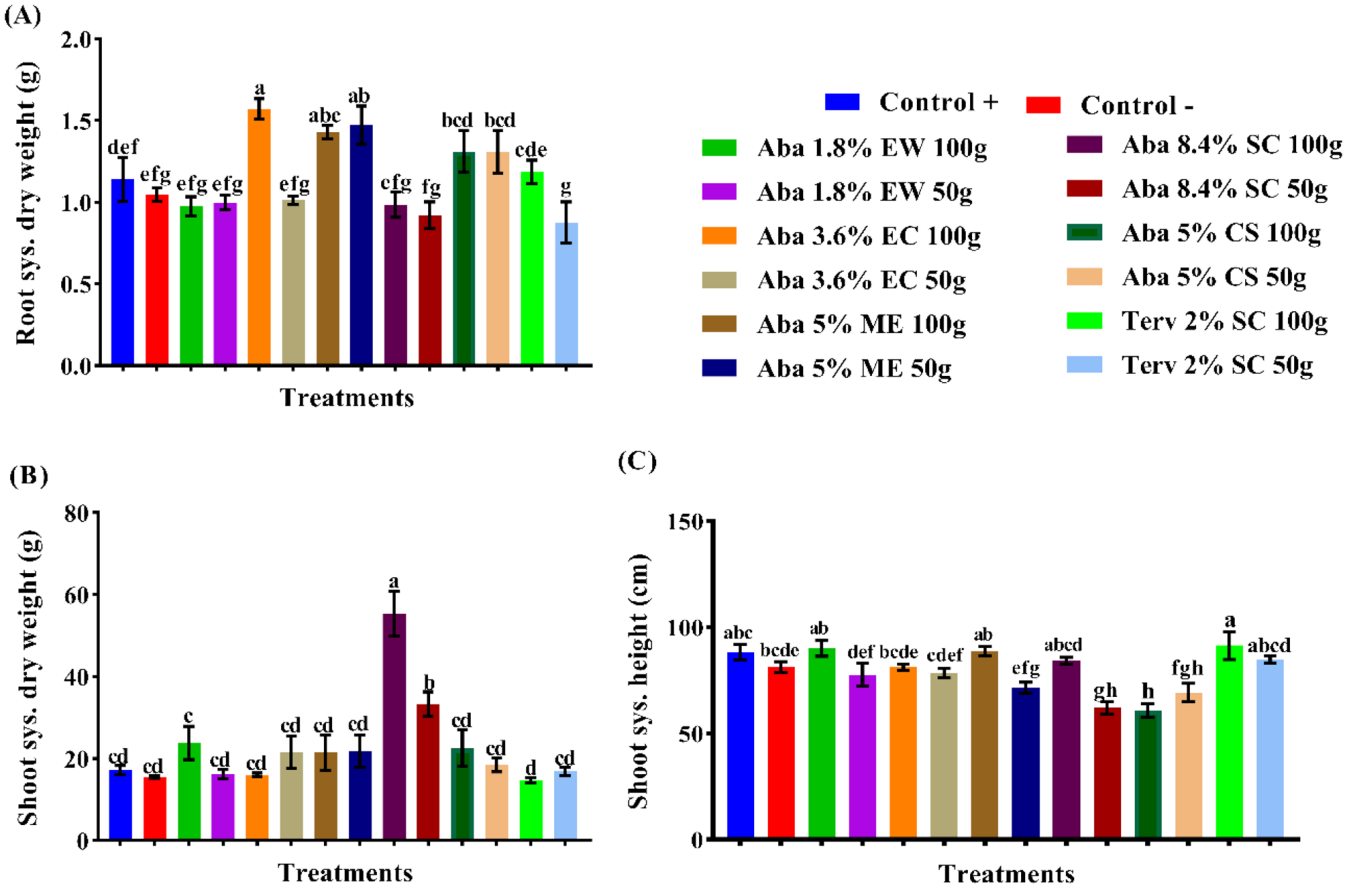
The efficacy of abamectin in various formulations and rates on root dry weight, shoot height and shoot dry weight of cucumber plants. (Means in each column followed by the same letter(s) are not significantly different according to LSD (*p* = *0.05*), values are means ± SEM).

**Figure 7:**
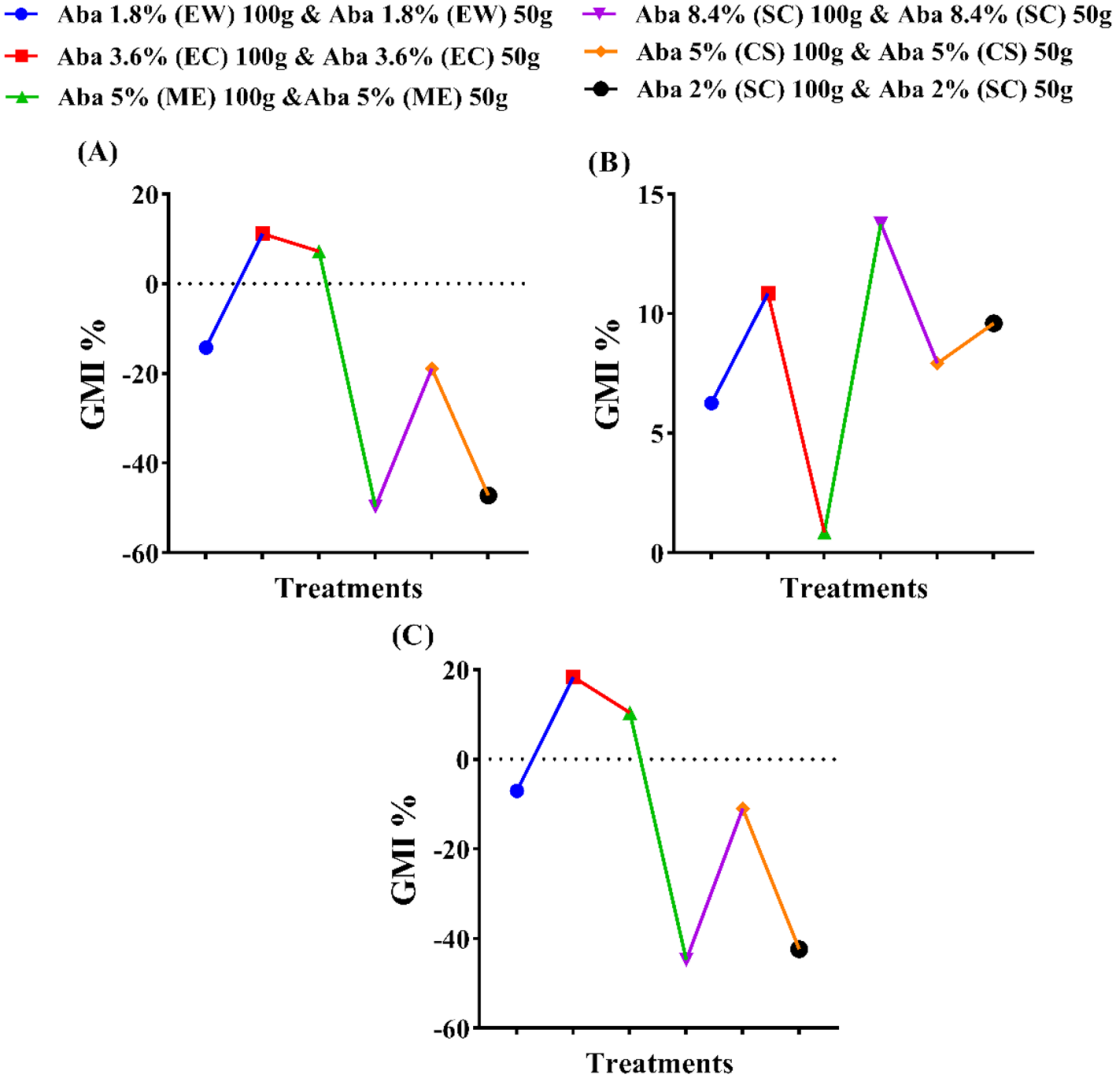
The influence of certain abamectin formulations at two rates as soil treatments on the root length, root radius and root surface area infected by (*Meloidogyne* sp.) on cucumber plants. (**A**) General mean increases (%) of the root length (m), (**B**) General mean increases (%) of the root radius (m), and (**C**) General mean increases (%) of the root surface area (m^2^). While GMI (%): general mean increases percentage.

**Figure 8:**
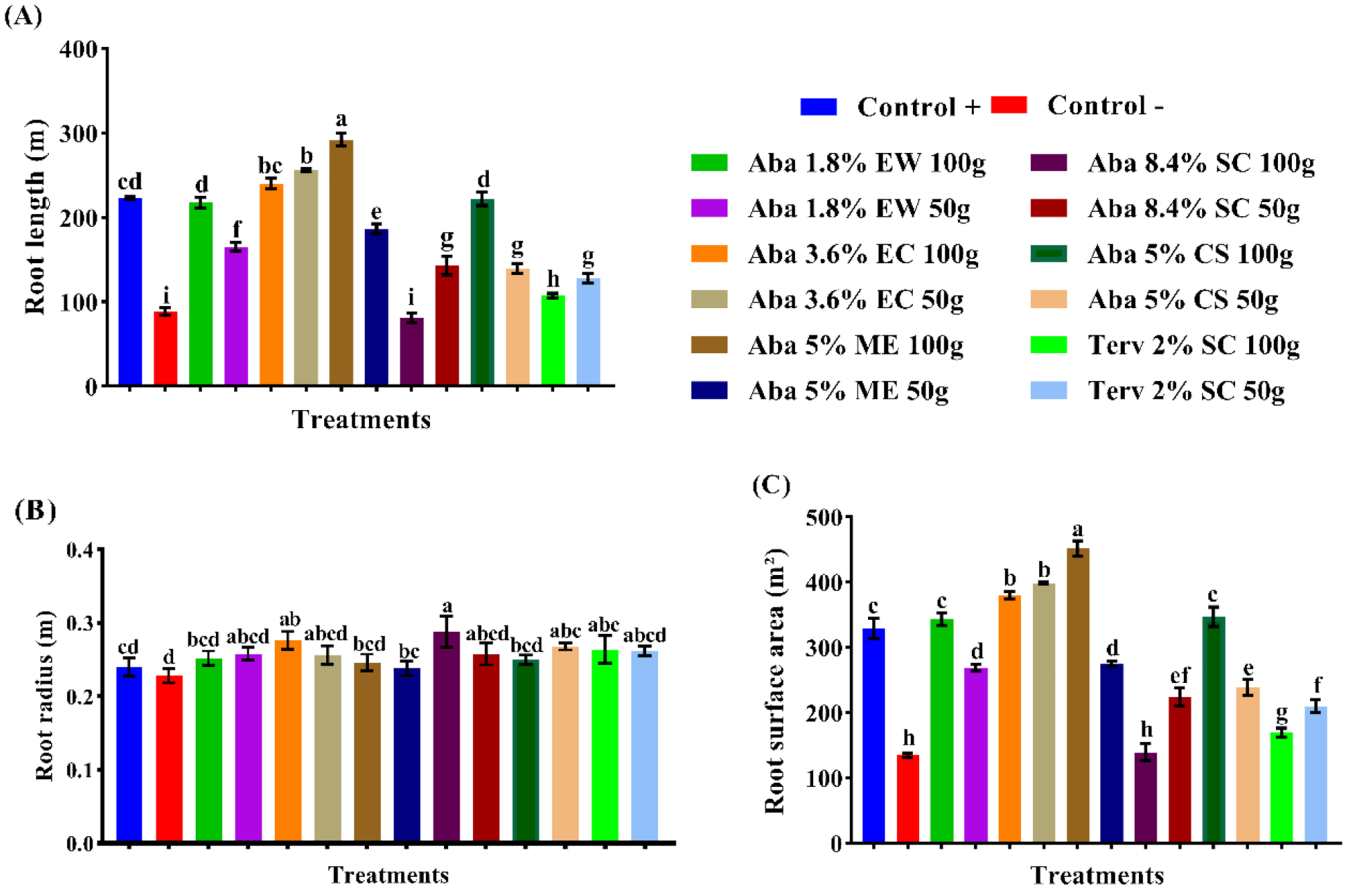
The efficacy of abamectin in various formulations and rates on root length (m), root radius (m) and root surface area (m^2^) of cucumber plants. (Means in each column followed by the same letter(s) are not significantly different according to LSD (*p* = *0.05*), Values are means ± SEM).

The influences of applied abamectin at different formulations on the growth parameters of cucumber plants were recorded (Figs. 5A–C, 6A–C, 7A–C, 8A–C). The recorded plant growth parameters were root dry weight, root length, root radius, root surface area, shoot dry weight and shoot height. In the untreated (uninoculated) plants, the root dry weight was decreased by 8.45%; also, the shoot dry weight and height were decreased by 9.60 and 7.99%, respectively (Figs. 5, 6). The obtained results showed that formulations of ME, CS and EC were the only treatments that recorded general mean increases in root dry weight of 27.22, 14.76 and 13.32%, respectively. While abamectin at SC (8.4%), EW and SC (2%) were decreased the root dry weight by 16.44, 13.40 and 9.68%, respectively. No significant differences were noticed between the high and low rates of EW, ME, SC (8.4%) and CS formulations. Meanwhile, all the formulations of abamectin such as SC (8.4%), ME, CS, EW, and EC, increased the shoot dry weight by 158.71, 26.20, 19.56, 16.65 and 9.69%, respectively, while SC (2%) reduced it by 7.67%. Unfortunately, there are no significant differences were observed between EW, EC, ME, CS and SC (2%) formulations at either high or low rates. Vice versa, all applied formulations of abamectin were minimized the shoot height of cucumber plants by 26.35, 17.17, 9.52, 9.24, 5.05 and 0.17% with CS, SC (8.4%), EC, ME, EW, and SC (2%), respectively. Application of abamectin at EC, CS and SC (2%) showed no significant differences between the high and low rates, while there are significant differences between EW, ME, and SC (8.4%) formulations (Figs. 5, 6).

The efficacy of abamectin formulations in the presence of root-knot nematodes were evaluated on the root length, root radius and root surface area (Figs. 7A–C, 8A–C). The application of abamectin in EC and ME formulations increased the root length by 11.19 and 7.24%, successively. Otherwise, SC (8.4%), SC (2%), CS and EW formulations decreased the root length by 49.77, 47.25, 18.91 and 14.23%, successively. In the same context, the root surface area was increased with EC and ME formulations by 18.37 and 10.45%, consecutively. While the remaining treatments, namely SC (8.4%), SC (2%), CS and EW formulations, diminished the surface area of cucumber plants by 44.80, 42.37, 11.00 and 7.02%, consecutively. The abamectin formulation of EC exhibited no significant differences between the higher and lower rates for both root length and root surface area. The root radius of cucumber plants was increased by 13.75, 10.84, 9.59, 7.92, 6.25 and 0.84% with SC (8.4%), EC, SC (2%), CS, EW, and ME formulations, consecutively. Moreover, all the tested formulations of abamectin exhibited no significant differences between the higher and lower rates.

### The impact of abamectin formulations on macro elements in cucumber roots

**Figure 9:**
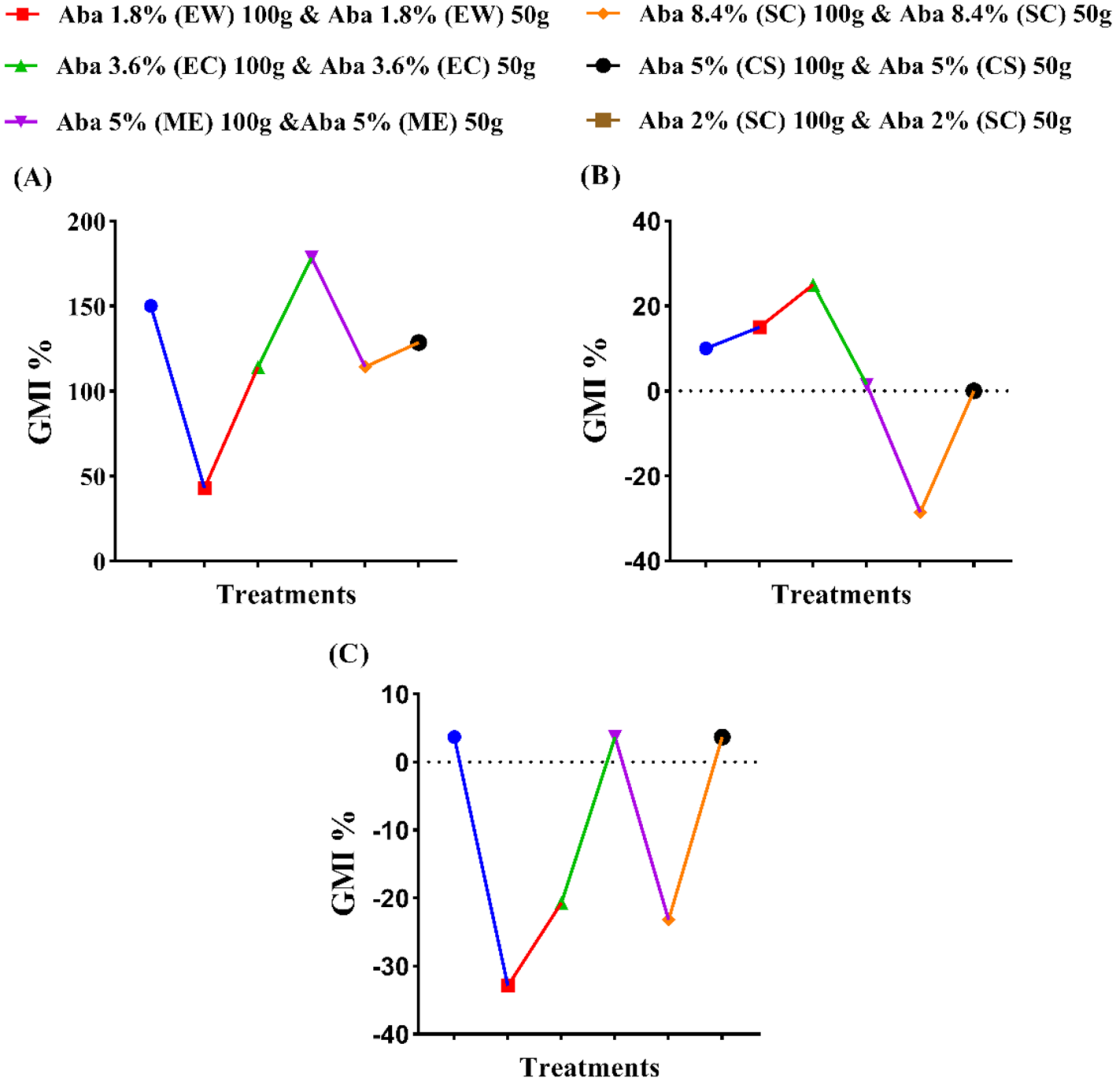
The influence of certain abamectin formulations at two rates as soil treatments on content of Nitrogen (N), phosphorus (P) and potassium (K) elements in cucumber infected by (*Meloidogyne* sp.). (**A**) General mean increases (%) of Nitrogen (N), (**B**) General mean increases (%) of phosphorus (P) and (**C**) General mean increases (%) of potassium (K). while GMI (%): general mean increase percentage.

The effects of infection with the root-knot nematodes and different formulations of abamectin on the content of nitrogen (N), phosphorus (P) and potassium (K) elements in cucumber roots were measured as found in (Fig. 9). The obtained results indicated that the untreated (uninoculated) pots had increases of N and P by 78.57 and 20.00%, respectively. The content of K was decreased by 22.00%. The macro element analysis exhibited that the content of N was increased with the formulation of SC (8.4%), EW, SC (2%), CS, ME, and EC by 178.60, 150, 128.60, 114.30, 114.30 and 42.86%, sequentially. The applied formulations, as ME, EC, EW, and SC (8.4%), were increased the content of P by 25, 15, 10 and 1.5%. However, SC (2%) did not achieve any increase (0.00%), while the application of CS formulation decreased the content of P by 28.50%. Differently, the content of K was enhanced by 3.70, 3.70 and 3.70% with EW, SC (8.4%) and SC (2%), sequentially. In contrast, the application of ME, CS and EC formulations minimized the content of K by 20.70, 23.20 and 32.90%, respectively.

## Disscusion

During the current discussion, we present the main effect of root-knot nematode on cucumber plants and the use of the abamectin formulations as an alternative tool to control the *Meloidogyne* spp. Root-knot nematode is widely distributed in greenhouses of cucumber production in Egypt. The idealistic root galling symptoms were noticed in either the absence of nematicides or as a result of control failures as reviewed by^34^. Currently, in Egypt, there are few of available options for managing *Meloidogyne* sp. and the non-chemical control agents are commercially difficult to be available and often unsatisfactory^35^. During this study, various concentrations of abamectin at different formulations, namely, SC, EW, ME, EC and CS, exhibited different levels of mortality which could be due not only to the active ingredient concentration but also to the adjuvants in the examined products, and these findings agree with^36^.

In accordance with other studies, it was stated that the formulations of abamectin are the key factor in the biological activity against plant parasitic nematodes^23^. Other study by^37^ reported that abamectin in the SC formulation is more effective than EW under laboratory conditions. Also,^38^ clarified that abamectin as SC at certain doses decreased the soil population of *M. incognita* infested tomato plants at a range of 23.40–43.29%, while abamectin as EW recorded a reduction at a range of 25.67–34.37%. However, no phytotoxicity was detected for both formulations.

A remarkable reduction in soil population and root gall index of *M. incognita* was achieved with abamectin (2.5% EC) and cadusafos under pot or field conditions^39^. In pot experiments, abamectin (VERTIMEC 1.8% EC) at 100 and 200 µg/ml against *M. incognita* on cabbage plants cv. Balady were minimized the galls, which ranged from 40 to 88%, while egg masses ranged from 58 to 98% and these are data in the same line as our results^40^.

In the same context, the superiority of abamectin as SC against the final populations in compared with the remaining formulations may be attributed to the moderate adsorption ratio on soil particles, whereas, the EC formulation had the highest adsorption ratio, which dramatically decreased their mobility in soil^23^. The use of formulations as water-based suspension concentrate (SC) provided environmental, economic, and social advantages, which included the safety to the applicators and the environment, ease of handling, relatively low cost, a high concentration of insoluble active ingredients, and the ability to be built in water-soluble adjuvants for enhanced biological activity^41^.

Meanwhile, Radwan^42^ elucidated that abamectin (SC) or emamectin benzoate (WDG) showed high toxicity against the J_2_ of *M. incognita *in vitro. Emamectin and abamectin were succeeded in decreasing galls, egg masses, eggs, and soil population density significantly. The use of abamectin (2% SC) alone is more effective than the binary mixture with *Trichoderma album* against *M. incognita* in soil or in tomato roots. The same trend was recorded with the number of egg masses, produced eggs, and females^1^.

It's worth mentioning that our obtained results indicate that some applied formulations of abamectin showed a negative effect on cucumber growth and the content of phosphorus and potassium elements, but many researchers find the opposite. On the other hand, the cucumber yield and plant height had increased significantly with abamectin (5% EC) at low or high rates compared with a crop produced without using abamectin**.** However, using dazomet or chloropicrin in combination with abamectin exhibited no significant differences in the total crop yield^43^. The tomato fresh weight and height showed the same significance with untreated check when applied abamectin SC or EW^38^. The application of abamectin (2% SC) achieved increases in the shoot and root dry weights of tomato plants by 16.92 and 14.26%, respectively^42^. The total marketable yield of tomatoes and the plant growth were improved when applied abamectin (2.5% EC) and cadusafos were applied against* M. incognita* at 5, 7.5 and 10 L/ha^39^. Also, the growth of olive plants e.g., the fresh weights and the length of both shoot and root, were increased at a range of 15.5 up to 105.8% over control with application of abamectin against *M. incognita* in compared with three tested bio-agents under greenhouse conditions^44^.

The pesticides that used during crop production processes inevitably remain in the soil, affecting rhizosphere microorganisms and plant growth as reviewed by^45^. However, during the current study, some treatments of abamectin formulations exhibited decreasing in the growth parameters for cucumber plants and this may be attributed to the residue of abamectin and/ or its metabolites which adversely affect the soil invertebrates and the roots of cucumber, and this finding is in the same line with those obtained by^43,46^. Furthermore, abamectin is degraded to 8a-hydroxyavermectin B1_a_ which is a low toxic product that may be taken up as a carbon source for microorganisms and then struggle with plant roots on nutrients^20,47^.

In conclusion, abamectin is an effective nematicide that has been recorded to control a wide range of plant parasitic nematodes, for instance, *Meloidogyne spp., Rotylenchulus reniformis* and *Tylenchulus semipenetrans*, on different crops in line with the global trend in integrating nematode management^48^. In current study, abamectin has shown a good efficacy in controlling the root-knot nematodes (*Meloidogyne* spp.). Also, the formulation as SC (8.4%) recorded the highest reductions in total population density (*J*_*2*_ + eggs), gall formation, and egg masses. However, the EW formulation was the least effective treatment. Current results recommend more research on abamectin formulations to determine their exact biological activity against plant parasitic nematodes under different environmental impacts and to achieve the prospective aims of sustainable agriculture.

## Data Availability

All data generated or analyzed during this study are included in this article.
